# Adapting teaching and learning in times of COVID-19: a comparative assessment among higher education institutions in a global health network in 2020

**DOI:** 10.1186/s12909-022-03568-4

**Published:** 2022-06-28

**Authors:** Dewi Ismajani Puradiredja, Linda Kintu-Sempa, Carola Eyber, Ralf Weigel, Bruno Broucker, Marie Lindkvist, Nuria Casamitjana, Rodney Reynolds, Hans-Friedemann Klinkel, Alberto Matteelli, Guenter Froeschl

**Affiliations:** 1 Bernhard Nocht Institute for Tropical Medicine, Hamburg, Germany; 2 Division of Infectious Diseases and Tropical Medicine, University Hospital, Ludwig-Maximilians-Universität, Munich, Germany; 3 Center for International Health, Ludwig-Maximilians-Universität, Munich, Germany; 4 Institute for Global Health and Development, Queen Margaret University, Edinburgh, UK; 5 Witten/Herdecke University, Witten, Germany; 6 Antwerp Institute of Tropical Medicine, Antwerp, Belgium; 7 Department of Epidemiology and Global Health, Umea University, Umea, Sweden; 8 ISGlobal, Hospital Clínic - Universitat de Barcelona, Barcelona, Spain; 9 High Point University, High Point, USA; 10 Institute of Tropical Medicine and International Health, Charité – Universitätsmedizin Berlin, Berlin, Germany; 11 Department of Clinical and Experimental Sciences, University of Brescia, and Spedali Civili, Brescia, Italy

**Keywords:** Higher education, Preventive measures, International health, Global health, COVID-19

## Abstract

**Background:**

This research examines the ways in which higher education institutions (HEIs) across the tropEd Network for Education in International Health (tropEd) began to adapt their teaching and learning approaches in response to the COVID-19 pandemic in 2020. Already during this early phase of the pandemic HEIs’ responses demonstrate global health approaches emphasising cooperation and communication, rather than national health driven strategies that emphasise quarantine and control. Key lessons learnt for multiple dimensions of teaching and learning in global health are thus identified, and challenges and opportunities discussed.

**Methods:**

Data collection includes a cross-sectional online survey among tropEd member institutions (*n* = 19) in mid-2020, and a complementary set of open-ended questions generating free-text responses (*n* = 9). Quantitative data were analysed using descriptive statistics, textual data were analysed using a Framework Analysis approach.

**Results:**

While early on in the pandemic the focus was on a quick emergency switch to online teaching formats to ensure short-term continuity, and developing the administrative and didactic competence and confidence in digital teaching, there is already recognition among HEIs of the necessity for more fundamental quality and longer-term reforms in higher education in global health. Alongside practical concerns about the limitations of digital teaching, and declines in student numbers, there is a growing awareness of opportunities in terms of inclusivity, the necessity of cross-border cooperation, and a global health approach. The extent to which the lack of physical mobility impacts HEI programmes in global health is debated.

**Conclusion:**

The COVID-19 pandemic has brought about preventive measures that have had a considerable impact on various dimensions of academic teaching in global health. Going forward, international HEIs’ experiences and response strategies can help generate important lessons for academic institutions across different settings worldwide.

**Supplementary Information:**

The online version contains supplementary material available at 10.1186/s12909-022-03568-4.

## Background

The first outbreak with the novel coronavirus (later named SARS-CoV-2) was reported in Wuhan, China in December 2019. On 30 January 2020, the WHO declared the outbreak of the new coronavirus as a Public Health Emergency of International Concern and defined COVID-19 as a pandemic on March 11, 2020 [1]. As of August 2021, the COVID-19 pandemic has resulted in over 198 Million confirmed cases and more than 4.2 Million deaths worldwide [2].

During the early phase of the pandemic it soon became evident that one effective preventive measure against the transmission of SARS-CoV-2 is physical or social distancing [3]. Therefore, many countries implemented temporary lockdowns from March 2020 onwards, and closed their higher education institutions (HEIs) [4, 5]. These sudden changes had a considerable impact on teaching and learning at HEIs worldwide.

However, while extant surveys and polls show the impact the COVID-19 pandemic has had and is having on the higher education sector at the aggregate level (e.g., QS Survey), they tend to lack more context-specific data for specific disciplines, such as international and global health, which due to their trans-disciplinary and global nature are intrinsically associated with mobility. Moreover, recently there have also been calls to “re-imagine global health teaching” more fundamentally [6, 7], such as in terms of global cooperation and solidarity [8, 9]. However, structured survey research tools alone tend to be ill-equipped to capture these important yet less quantifiable perspectives and sentiments.

**Fig. 1:**
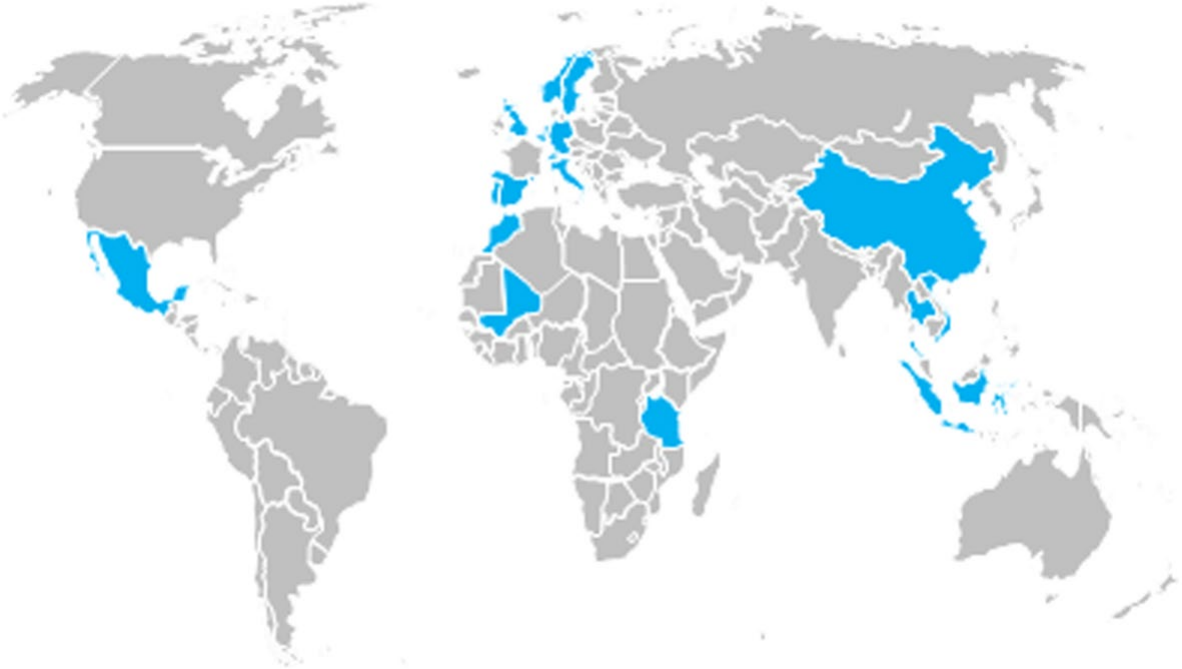
World map with member and participant countries

In response to these recent calls to reform global health teaching, this study synthesises and compares both quantitative and qualitative insights from senior academic staff actively involved in global health teaching at 20 higher education institutes across the tropEd Network for Education in International Health (tropEd) on four continents: Europe, Africa, Asia, and Latin America. TropEd as it is known today, was established in 1996 with the goal to connect global health institutions and offer a joint framework for internationally accredited master’s degree programs in the field of international/global health. It is currently the largest network for a master’s degree in international/global health with 28 HEI in 17 countries (Fig. 1). The tropEd members govern the Network and collaboratively establish quality assurance standards for global health education, accredit courses offered by member institutes, and develop pedagogical practices that intend to improve global health postgraduate education [10, 11].

Although the tropEd Network’s member institutions all specialize in global and international health, the ways in which they approach teaching and learning in relation to the topic and practice of infectious disease control vary broadly in nature and scope. In addition, at the time the study was conducted member institutes found themselves in varying situations depending on the intensity of their local COVID-19 epidemic and the type of national response taken. Thus, the experiences of such an international consortium of institutions offer great benefits for generating insights into how universities in different context have confronted and adapted their international/global health teaching and learning in response to the COVID-19 pandemic.

This paper highlights the experiences of HEIs within the context of the COVID-19 pandemic across a collaborative and diverse global education network with a special focus on master’s level education in global and international health – a discipline typically characterised by a high degree of international and inter-institutional student and staff mobility. More specifically, the objectives are to (i) describe and compare both quantitative and qualitative data on the impact and response to COVID-19 among higher education institutions in a global health network in the early phase of the pandemic in 2020, (ii) to explore anticipated challenges and opportunities for adapting teaching and learning approaches in global health at the time, and, (iii) and, to explore and discuss the implications of these challenges for a global higher education network, such as tropEd.

## Methods

### Data and sampling

Between 4 May to 1 June 2020, senior academic staff involved in international and global health teaching at the 28 tropEd member institutions from 17 countries on 4 continents (see Fig. 1) were invited to complete a 17-item online survey questionnaire (see Supplement 1). The survey included questions regarding the tropEd member institutions’ strategies in response to COVID-19, and their concerns at the time. Alongside the cross-sectional online survey among tropEd member institutions, a complementary set of open-ended questions (see Supplement 2) generated free-text responses, which respondents reported in the form of Microsoft Power Point presentations. One representative per tropEd member institute could submit a response to the survey and set of open-ended questions respectively to avoid skewed results. Out of the 28 eligible respondents, 19 completed the survey questionnaire, and 9 submitted a response to the set of open-ended questions, resulting in return rates of 19/28 (67.9%) and 9/28 (32.1%) respectively.

 The world map shows in blue the locations of the 28 tropEd member institutions in 17 countries. Member institutions from all countries except Mali and Indonesia participated in the study. (world map is based on an in-house template)

### Analyses

Quantitative data were summarised and analysed using descriptive statistics (frequencies and proportions) via a database and statistical software (Microsoft Excel 2010; Stata SE 15).

Textual data were organised using Microsoft Excel and analysed using a Framework Analysis approach [12]. At first the free-text responses were iteratively read and coded by LKS and DIP independently. In intermittent discussions LKS and DIP agreed on a set of codes and grouped codes that were conceptually related into thematic coding categories. This represented the analytical framework. The coded data were then summarised in a matrix. Both a priori themes guided by the research objectives as well as new themes were generated from the data. A priori themes explored included: general COVID-19 impact at the institutional level; direct impact on teaching and learning activities, as well as on immediate students and staff; and, an outlook. New themes that were generated inductively relate e.g., to the anticipated challenges and opportunities discussed, and include e.g., opportunities for ‘greater inclusion’ and the potential for ‘reforms in teaching and learning’.

Summaries of written responses to the open-ended questions are presented in the results organised by themes, and representative quotes have been selected from a variety of respondents in order to avoid selectivity in the range of data presented. Respondents are identified by the institution they are affiliated with, rather than their personal characteristics, and their quotes referred to as follows: (Institution, Country of institution, Power Point Presentation (PPT) slide number or open Survey Question (SQ) number), for example Bernhard Nocht Institute for Tropical Medicine (BNITM), Germany, PPT, s. 3.

### Ethical considerations

Participants in this study were all representatives of member institutions of the tropEd Network for Education in International Health, who were also participating in the conception of the study. Participants’ consent to participate and publish the findings from the survey in a peer-reviewed journal was sought through a written informed consent form. Participation in the survey was voluntary and participants were able to withdraw freely from participation in this study at any point in time and without having to give any reasons. Participants were given the explicit option to decline answering questions without having to give any reasons. The institutional review board, the Ethics Committee of the Ludwig-Maximilians-Universität Munich, ruled that no formal ethics approval was required, given the nature of the study, which highlights COVID-19 related impacts and procedures with regard to higher education institutions. All methods were carried out in accordance with the Helsinki Declaration and further relevant guidelines and regulations.

## Results

In the following, we firstly describe the immediate impact and response strategies of higher education institutions in global health (study Objective i). Furthermore, we will highlight the challenges and opportunities institutions, students, and staff were experiencing and anticipating during the early phase of the pandemic (study Objective ii), as well as in terms of implications for higher education networks, such as tropEd (study Objective iii).

Table 1 shows those institutes of those respondents who participated in the online survey (*n* = 19) and of those who responded to the additional open-ended questions (*n* = 9).

**Table 1:** List of participating tropEd institutions

Participating tropEd Member Institutions	Survey questionnaire (*n* = 19)	Complementary set of open questions (*n* = 9)
AFRICA		
Ecole Nationale de Santé Publique, Morocco	X	
Ifakara Health Institute, Ifakara, Tanzania	X	
AMERICAS		
National Institute of Public Health, Mexico City, Mexico	X	
ASIA		
Fudan University, China		X
Khon Kaen University, Thailand	X	
University of Public Health, Hanoi, Vietnam	X	X
EUROPE		
Institute of Tropical Medicine, Antwerp, Belgium	X	
Charité – Universitätsmedizin Berlin, Berlin, Germany	X	X
Bernhard Nocht Institute for Tropical Medicine, Hamburg, Germany	X	X
Institute of Global Health, Heidelberg, Germany	X	
Center for International Health, Munich, Germany	X	X
Witten Herdecke University, Witten Herdecke, Germany	X	
University of Brescia, Italy	X	X
University of Bergen, Norway	X	
Instituto Higiene e Medicina Tropical, Lisboa, Portugal	X	
Institute for Global Health, Barcelona, Spain	X	X
Umea University, Sweden	X	X
Swiss TPH, Basel, Switzerland	X	
Royal Institute of Tropical Medicine, Amsterdam, The Netherlands	X	
Queen Margaret University, Edinburgh, UK	X	X

Continents, countries and cities are listed in alphabetical order

### Findings from the structured survey

#### Impact and concerns during the early phase of the COVID-19 pandemic

The immediate impact of the protective measures taken against the spread of COVID-19 was the sudden necessity to find alternatives to in-person teaching modalities. Indeed, all of the 19 institutions that participated in the survey declared that their institution had to adapt to distance learning either much (9/19; 47.4%), or very much (10/19; 52.6%).

Representatives of member institutions were asked about their worries with regard to the pandemic. The following table (Table 2) shows the numbers of institutions that declared the respective issues as “much” or “very much concerning”.

**Table 2:** Concerns by continent

	Africa *n* = 2	Americas *n* = 1	Asia *n* = 2	Europe *n* = 14 (%)	Total *n* = 19 (%)
Staff mobility	1	1	1	5 (35.7%)	8 (42.1%)
Student mobility	1	1	1	8 (57.1%)	11 (57.9%)
Staff and student health	1	1	1	10 (71.4%)	13 (68.4%)
Digital transformation	1	1	2	6 (42.9%)	10 (52.6%)

Major concerns as perceived by institutional representatives. Multiple answers were possible. Proportions are given where meaningful

#### Response strategies during the early phase of the COVID-19 pandemic

All but one institution stated “switching to online formats” a key strategy (92.9%), while eight cancelled some courses (42.1%) and three offered new courses (15.8%). Of the 19 responding institutions, eight delayed the start dates of some courses (42.1%) whereby of these four moved some practical (lab based or experiential) courses to the next semester (21.1%). Some institutions temporarily made adaptations to their application process, such as changing application and acceptance deadlines (3/19; 15.8%), or adapted the selection process for new students (3/19; 15.8%). Two institutions had students completing their courses at a tropEd partner institution (10.5%). Two institutions modified their recruitment process (10.5%). Table 3 is outlining the strategies, stratified by geographic region.

**Table 3:** Response strategies by continent

	Africa *n* = 2	Americas *n* = 1	Asia *n* = 2	Europe *n* = 14 (%)	Total *n* = 19 (%)
Switch to online formats	2	1	2	13 (92.9%)	18 (94.7%)
Delay of course delivery			2	6 (42.9%)	8 (42.1%)
Delay in admission	0	0	2	2 (14.3%)	4 (21.1%)
New courses developed	0	0	1	2 (14.3%)	3 (15.8%)
Courses cancelled	0	0	2	6 (42.9%)	8 (42.1%)
Modify participant recruitment	0	0	1	2 (14.3%)	3 (15.8%)
Refer participants to partner institutions	0	0	0	2 (14.3%)	2 (10.5%)

Response strategies among tropEd member institutions by continent in 2020. Multiple answers were possible. In the table, the responses on “delay in application deadline” and “delay in acceptance delay” were combined resulting in “delay in admission”. The responses on “modify recruitment” and “adapt selection” were combined resulting in “modify participant recruitment”. Proportions are given where meaningful.

#### Digital infrastructure at the beginning of the COVID-19 pandemic

The participating institutions described the preparedness of their institutions in terms of digital infrastructure (online tools, but also capacitation of the teachers) to meet challenges that arose in the COVID-19 pandemic in 63.2% (12/19) as “much prepared” and in 15.8% (3/19) as “very much prepared”. Only 3 institutions in Europe declared their institutions to be “less” or “not at all prepared” (15.8%).

#### Outlook

In 11 out of 19 institutions the representatives were willing to make an attempt at a forecast on the development of student numbers as an impact of the pandemic. The representatives expressed fear of decreasing student numbers in 5 out of 11 institutions (45.5%), here all Asian institutions were concerned as well as half of the responding European institutions. In two institutions the student numbers were expected to remain at pre-pandemic levels, and in four institutions (36.4%) an increase in student numbers was expected in the post-pandemic future.

### Findings from the open textual responses

A priori and new themes are summarised in Table 4, along with the regional context within which these were raised by respondents. Individual aspects relating to these themes are presented under the main topics ‘impact’, ‘outlook’, and ‘implications for tropEd Network’ are presented below.

**Table 4:** Themes in the final analytical framework and the regional context within which the themes were raised

Themes	Regional context			
	Africa	Americas	Asia	Europe
COVID-19 impact				
At institutional level				
-Staff and resources diverted away to COVID-19 response			X	X
-COVID-19 research prioritisation				X
-Interruption/cancellation of research, teaching and capacity-building activities at partner institutions/sites overseas, particularly in low- and middle-income countries				X
On educational activities				
-Short notice transfer from face-to-face to online	X	X	X	X
-Cancellation of fieldwork-based activities				X
-Changes in student applications				X
On students				
-Switch to online teaching positively received				X
-Feeling overwhelmed by online content			X	
-Unstable internet connectivity		X		X
-Restricted mobility for international students/isolation			X	X
-Active engagement in COVID-19 response			X	X
On staff				
-Open/cooperative towards switch to online teaching formats				X
-Feeling apprehensive towards switch to online formats				X
-High work load and staff scarcity			X	X
Outlook: Challenges and opportunities				
-Administrative and didactic preparedness	X	X	X	X
-Innovation and reform in teaching			X	X
-Student intake and financial stability				X
-Social consequences			X	X
-Lack of physical mobility/presence	X		X	X
-Importance of health protection measures at HEIs			X	X
Implications for higher education network in global health (tropEd)				
-Capitalise on networking opportunities to share experience and resources			X	X
-Opening up to virtual solutions	X			X
-Promote inclusivity				X
-Communication and student representation				X
-Promote awareness of global health			X	X
-Advocate for solidarity	X	X		X

### Topic 1: COVID-19 impact during the early phase of the pandemic

#### Impact at the institutional level

Despite variations in the way the pandemic developed and was being addressed in the respective countries of respondents’ institutions, there were a number of similar issues that arose. During the early emergency phase of the COVID-19 pandemic, a number of respondents described how the response to the pandemic meant that inevitably resources needed to be diverted away from teaching and capacity-building programmes and activities at the respective institutions (e.g., Center for International Health, Ludwig-Maximilians-Universität (LMU), Germany; PPT, s. 1; Bernhard Nocht Institute for Tropical Medicine (BNITM), Germany; PPT, s. 1). Four respondents mentioned that faculty staff who would have had usually been involved in teaching activities were called to the frontline to address the emerging pandemic:

"Some of our researchers/faculty are clinicians working at the University Hospital [and] some of our experts are part of assessing committees to health authorities in Spain and Catalonia"

- ISGlobal, Universitat de Barcelona (IS Global), Spain; PPT, s. 1

Three respondents highlighted that during this time COVID-19 funding and research were prioritised over teaching and capacity-building activities at their institutions.

"COVID-19 research activities are certainly a priority, which has meant that at an institutional level teaching and capacity-building that is not directly related to COVID-19 has taken a backseat"

- BNITM, Germany; PPT, s.1

This meant in some instances that non-COVID-19 research, and research activities in partner institutions abroad were paused.

"All capacity strengthening activities in the context of research projects in Africa have been postponed."

- IS Global, Spain, SQ 2

Indeed, one respondent voiced concern over the impact this can have on partners in low- and middle-income countries, in particular fragile states (Institute of Global Health & Development, Queen Margaret University (QMU), UK; PPT, s. 1).

#### Impact on educational activities

Against this general backdrop the institutions found themselves in a situation whereby educational activities that would have taken normally place in person, had to be rescheduled or cancelled, or switched to online teaching formats within a short amount of time.

“We used to deliver mainly face-to-face courses. Most of our regular programs and courses offer switched to online from Friday to Monday. This had a great impact on our faculty and students, and also on our team. Short courses for international students and capacity building trainings were postponed or cancelled.”

- IS Global, Spain SQ 2.

During this ‘emergency phase’ the focus needed to be generally on creating quick alternatives to deliver content to students, and little room to adapt didactic approaches more thoroughly:

"The immediate strategy was to move delivery online and maintain content and delivery style"

- QMU, UK; PPT, s. 2

Similarly, other activities, such as data collection in the field in fulfilment of on-going master’s theses needed to be postponed.

While several of the respondents mentioned that their institutions feared a decline in student applications and indeed, one institution mentioned that they had applied for financial support from the government to compensate for loss in student fees due to a drop in applications, one institution reported an increase in the number of applications, perhaps as a result of an increased interest in/more visible relevance of global health education.

#### Impact on students and staff

The sudden changes due to COVID-19 affected students and staff in various ways. Some respondents reported that a number of their students were volunteering to help with the COVID-19 response (e.g., LMU, Germany, PPT, s. 3; University of Brescia, Italy; PPT, s. 3) either at COVID-19 Testing Units or in the form of “frontline health care work with high stress levels (QMU, PPT, s. 1). While three respondents reported that their students generally appreciated that their degree programme continued digitally and virtually/online, these changes were not without problems.

While some respondents reported that they had the impression that “participants are more focused” (Charité – Universitätsmedizin Berlin (Charité Berlin), Germany, PPT, s. 3) and “engaged during the online sessions – sometimes even more than during face-to-face teaching” (BNITM, PPT, s. 3), one respondent explained that students reported to be at times overwhelmed by the sheer amount of online materials provided to them – highlighting the need for more fundamental changes to online teaching formats:

"Many students complained they had so much video of different course to watch in advance. There [is] nearly no time left for them to think and review what they had learn. And some students even couldn‟t finish the video watching pre-class. That make them hardly to be involved or follow the discussion in web-class."

- School of Public Health, Fudan University (Fudan University), China; PPT. s. 3

On a more technical note, a number of respondents raised the concern over unstable internet connectivity (e.g., Umeå University, Sweden; PPT, s. 3; Charité Berlin, Germany; PPT, s. 3). One respondent described how local students returned to their homes within the country during the lockdown – some of them to rural areas with slow internet connection, which made it harder for them to live stream online lectures (National Institute of Public Health Mexico, Mexico; SQ. 3).

As a consequence of COVID-19 measures, such as pending travel restrictions, some respondents reported that a considerable number of international students decided to return to their home countries, while others remained “stranded in halls of residence” (QMU, UK; PPT, s. 1). This led to concerns for the well-being, in particular of international students. As travel restrictions were gradually implemented in different countries institutions, there were reports of students not being able to return home (Department of Epidemiology and Global Health, Ume University [Umeå University], Sweden; PPT, s. 3) or new incoming students would not be able to enter the country:

“International students from Laos and Cambodia cannot return to campus due to border lockdowns”

- Hanoi University of Public Health (HUPH), Vietnam; PPT, s. 1

Thus, in addition to implementing the necessary COVID-19 related social distancing and hygiene measures, three respondents also mentioned that their institutions offered psychological and social support to help students address problems that arose as part of the pandemic measures taken.

On the teaching side, while some respondents described lecturers as being generally open and cooperative to transfer to online teaching (e.g., LUM, Germany; PPT, s. 3), others seemed more apprehensive to see these changes in teaching strategies/here too challenges relating to the amount and nature of online communication were being described (e.g., QMU, UK; PPT, s. 3).

Further, depending on the intensity of the epidemic in their respective countries, a number of lecturers needed to be actively involved in the COVID-19 response, e.g., by working in hospitals at the time (e.g. University of Brescia, Italy; PPT, s. 3), or they were concurrently involved in national steering committees to address COVID-19 (e.g. HUPH, Vietnam; PPT, s. 3), highlighting the burden of adapting educational activities on short notice, competing COVID-19 work assignments, and a reduced availability of teaching staff. As one respondent described it:

“[…] the limited availability of our staff: it is a lot of work to change the teaching. We are all working many hours to keep things going and to adapt."

- KIT Royal Tropical Institute (KIT), Amsterdam, Netherlands, SQ 13.

At the same time, the increased use of online teaching and learning formats facilitated the recruitment of lecturers in places because it does not require travel and is hence ‘time efficient’ (Charité Berlin, Germany; PPT, s. 3).

### Topic 2: Outlook: Challenges and opportunities

#### Administrative and didactic preparedness

The uncertainty about how the pandemic and measures to address it would evolve, raised a number of questions. Alongside the need to implement adequate health projection measures (Khon Kaen University, Thailand, SQ 7; HUPH, Vietnam, SQ 7; Instituto Higiene e Medicina Tropical, Portugal, SQ 7), some respondents (e.g., IS Global, Spain; SQ 2; Antwerp Institute of Tropical Medicine, Antwerp (ITM Antwerp), Belgium, SQ 7)) highlighted that institutions needed to plan for different scenarios for the upcoming academic year depending on travel restrictions, and decide and prepare their teaching modalities accordingly (i.e. online, face-to-face and/or hybrid).

[We are worried about] the uncertainty of what is going to happen in the next academic year. Our Master program is face-to-face with 70% students from abroad. Now, we need to plan for face-to-face, online and blended scenarios, because we do not know what is going to happen. The recruitment of students (mainly international) is very difficult in this context for economic and non-travel policy issues.”

- IS Global, Spain, SQ 3.

In particular, transferring longer and practical courses into online formats was being described as challenging, as well as establishing fair and just online exam procedures (e.g., Ifakara Health Institute, Tanzania, SQ 2). At the same time the academic schedule that had in part shifted due to course cancellation and postponements earlier in the year, would need to be caught up on. Inevitably, adapting teaching and learning formats required time and resources, as well as building “administrative and didactic competence and confidence” (Charité Berlin, Germany, PPT, s.3).

#### Innovation and reform in teaching

Despite the practical challenges, a number of respondents also described the situation as a chance to develop innovative “new exciting lecture formats” (Charité Berlin, Germany, PPT, s. 3), and allowed for greater access and “participation in international webinars” (University of Brescia, Italy, PPT, s. 4). More fundamentally, digital and virtual teaching and learning approaches acquired by necessity could lead to positive changes in student–lecturer interpersonal power dynamics (Charité Berlin, Germany, PPT, s. 3), and facilitate more profound reforms in teaching and learning:

“A major challenge is the change in learning model from passive to active learning. Students used to sit in class, listen and “accept”, but now they [need to] learn online by themselves.”

- Fudan University, China; PPT, s. 3

Already during this early phase of the pandemic, respondents were considering to keep at least part of the newly introduced digital and virtual teaching modalities independently of the future development of COVID-19:

"The whole situation has given us opportunity to learn about online teaching and surely we will develop several online courses over the coming years."

- Umeå University, Sweden; PPT, s.4

#### Student intake and financial stability

At the time, it was also unclear how changes from face-to-face to online teaching and learning would lead to changes in student intake. Respondents were weighing up whether the COVID-19 pandemic would generate greater interest in the discipline of global health, and whether the possibility to study online would increase students numbers due to greater affordability, or lead to a drop in student numbers, especially of those coming from abroad, due to a lack of social interaction and first hand experience of different educational settings.

“Face to face meetings are important to create human and professional linkages. Students might be less interested in [tropical medicine] courses without the possibility to move out of their country.”

- University of Brescia, Italy; SQ 12.1

“Travel restrictions probably still persist at the beginning of next academic year or may be only lifted during the next academic year. This might result in students not being able to come to Berlin (or only later during the course). […] Students who do the Programme also because of the social interaction and because of the Berlin experience may postpone to 2021 (lower numbers next year?). On the other hand the online formats allows students who could not come to Berlin otherwise (and not necessarily only because of Covid-19) still to participate (increase numbers?)...”

- Charité Berlin, Germany, SQ 12.1

The latter would have implications in terms of financial stability, which was raised as a topic of concern.

“Changing face-to-face teaching to online formats is challenging and takes a huge effort, on the upside offering courses online could potentially give those students the opportunity to join, who could normally not afford to attend. However, this will of course have financial implications - what should the fees be for an online course? Should the fees be less but the number of students who can attend a course higher?”

- BNITM, Germany, SQ 12.1

#### Social consequences

Alongside the more practical and economic challenges of adapting teaching and learning, respondents were also concerned about the social consequences of the COVID-19 pandemic, in particular for students whose work and family situations are directly affected (QMU, UK, SQ 13), and highlighted the necessity of 'timely decision making and unequivocal communication with future students in a context of uncertainty’ (ITM Antwerp, Belgium, SQ 13). Furthermore, one respondent considered the possibility of compromised staff morale if work continues remotely:

"We [used to] travel, work on the ground - not anymore"

- QMU, UK; PPT, s. 4

#### Lack of physical mobility

Indeed, one of the key concerns raised related to the lack of physical mobility of student and lecturers, which are usually a central part of global health and its popularity as a discipline in general, and the tropEd master programme in particular.

"It's difficult to imagine an international campus without the physical presence of international students"

- ITM Antwerp, Belgium, SQ 13

This would have implications for the degree of traveling for thesis fieldwork and moving in between tropEd institutions, but also in terms of social interaction and networking activities both among students and members of the tropEd Network.

"After taking the hurdle of people applying and getting funding the next thing is the start: how to create a learning community with a lot of people not in class from the start?”

- KIT, Netherlands, SQ 13.

However, respondents also highlighted the opportunities of increased online teaching and learning.

Teacher mobility is relative, and actually the digital teaching formats encourage (digital) mobility of teachers. Online teaching is also saving teachers time and the institutions cost (for travel and accommodation) […]. A general dilemma in tropEd is that (physical) mobility is in conflict with ecology. We have to take ecology very serious and the current situation demonstrates that we can still provide good quality education without extensive (physical) mobility.

- Charité Berlin, Germany, SQ 12.1.

Further, the online teaching formats could help in making the global health masters programmes more accessible to people with limited resources:

"We consider learning online teaching formats as useful, not only should comparable scenarios arise, but also to perhaps offer more inclusive (distance learning) options for students who can otherwise not attend due to affordability."

– BNITM, Germany, PPT, s. 3

However, this would require more equitable and stable access to the internet, also for those students living in more remote areas (e.g., National Institute of Public Health Mexico, Mexico, SQ 3). Thus, increased online teaching and learning would need to be accompanied by increasing IT infrastructure, and ‘e-learning capacities’ (Ecole Nationale de Santé Publique (ENSP), Morocco, SQ 13). In particular, if existing international partnerships and courses are to be continued during the pandemic, one respondent proposed that overseas partners should be supported in building the necessary IT infrastructure (LUM, Germany, PPT, s. 4).

Overall, however, cautious optimism prevailed. As one of the respondents concluded:

“The impact might be felt as negative at first while we are still in the process of adaptation but it might turn out to be positive in the longer run...”

- BNITM, Germany, SQ 12.1

### Topic 3: Implications for a higher education network in global health: tropEd

Prior to the COVID-19 pandemic, the global connectedness of the tropEd Network’s member institutions depended to a significant extent on international travel and constant exchange with each other using digital resources. In contemplating the implications of the COVID-19 pandemic for tropEd Network, respondents highlighted the need to build on and further develop its existing vision and mission (e.g. QMU, PPT, s. 5) to promote greater inclusivity, for example, through holding general assemblies online (e.g. LMU, PPT, s. 5), and by ensuring transparent communication with and among students and greater representation, in particular of international students within the Network (e.g., University of Brescia, Italy, PPT, s. 5; BNITM, Germany, PPT, s. 5). Furthermore, respondents mentioned the need to advocate for solidarity, and to capitalise on tropEd’s networking opportunities to share experience, resources, and engage in collaborative initiatives (e.g., Umeå University, Sweden; PPT, s. 5; QMU, UK; PPT, s. 5). While respondents saw it as the Network’s continued mission to promote global health awareness, both in terms of teaching and learning content (e.g. HUPH, Vietnam; PPT, s. 5), but also in terms of enabling student and lecturer exchange and first-hand experience in the field and different educational environments, there was also a call for increased virtual mobility to reduce the Network’s carbon footprint (e.g. Charité Berlin, Germany, PPT, s. 5).

## Discussion

This study uses both quantitative and qualitative data to produce a comparative analysis of experiences of HEIs within the context of the COVID-19 pandemic across a collaborative and diverse global education network with a special focus on master’s level education in global and international health.

The quantitative findings show that in the early phase of the COVID-19 pandemic despite the necessity for immediate changes in teaching and learning modalities, and uncertainty about how the pandemic and measures to address it would evolve, higher education institutions across the tropEd Network showed to be resourceful and were able to adapt.

As most important and at the same time immediate measures the institutions stated digital transformation and cancellations of courses, which can be expected to also have direct impacts on student trajectories. It can be assumed that study periods will have to be extended. In addition, the sudden online delivery of course contents have been presenting a challenge to fulfilling learning objectives, especially in courses that are covering practical contents, such as laboratory-based formats. As a consequence, the evolution of digital transformation was perceived as a major concern at almost half of the institutions. However, of primary concern was the health of staff and students that was put at risk through educational in-person interaction.

As COVID-19 developed into a global pandemic, student mobility, which is a core element of study trajectories within the tropEd Network, came to a halt. As has been reported elsewhere, many international students were still able to return to their home countries before travel restrictions were implemented and all flights were cancelled [13]. However, others were left stranded either at their university or in third intermediate countries, which exacerbated social isolation, vulnerability, and unexpected economic hardship. International students who were able to return home faced various obstacles there. Even though most of the institutions in our study were able to offer online teaching for the immediate time period, some reported that poor internet service prevented many students from attending online seminars without difficulty, as has been reported in a review by Sahu [14]. Some of the students may have had financial disadvantages due to expensive internet services in order to be able to attend online classes, potentially increasing inequity in accessing higher education. The rapid reorganization from face-to-face teaching to online teaching might have overcharged some universities, potentially also aggravating inequities in the operations and as a consequence in reputation of academic institutions. On the other hand, online classes may provide an opportunity for students to manage their courses according to their own schedule, therefore providing flexibility and accessibility [15]. Especially for international students the uncertainty about what will happen next was pervasive. As has been reported elsewhere, many international students were worried about their own safety, about being stranded on closed universities and campuses or in hotels, or temporary accommodations that expose students and their families to increased economic vulnerability [13, 15]. Many HEI encountered a number of important challenges, including an initial lack of adequate digital infrastructure and digital competency of academic staff, the rapid reorganization from face-to-face teaching to online teaching, and reduced financial returns due to the loss of international students in upcoming semesters [16]. Through financial restructuring that invests significant parts of institutional budgets into COVID-19 research, disciplines and departments that cannot quickly adapt to their university’s new priorities may find themselves unable to pay their research staff or faculty [17–19]. This resource diversion was also reported by participating institutions in our study. As a consequence, researchers may expect to produce fewer publications and to have to adjust to reductions in funding and loss of staff resulting in reduced capacity for teaching. As an underlying structural deficit in some HEIs teaching staff is cross-financed by research revenue. Another associated challenge is recruiting new trial participants in the midst of a pandemic, which could cause long-term delays of many clinical trials [17], hence also creating difficulties in the longer run in finding suitable master thesis projects. For the fall semester 2020, fewer student applications were expected by our study participants to mean reduced income from tuition fees. As many universities depend on these fees to finance faculty salaries, benefits and research, these sources of lost or reduced income could have destabilizing effects for the HEI sector [20].

The qualitative findings show how already in the early phases of the pandemic, higher education institutions were considering not merely the technical, practical, and financial implications of adapting teaching and learning in global health, but were beginning to contemplate the more far-reaching, longer term consequences of the COVID-19 pandemic in the discipline of global health. The circumstances of the pandemic have certainly presented challenges from the start but also opportunities to (re-)evaluate and (re-)consider more fundamental issues [21]. One central theme is that of physical versus digital mobility, and concerns about ecology and inclusivity on the one hand, and social interaction and “lived experience” in diverse socio-cultural settings and educational environments on the other hand [7]. The situation of travel restrictions, quarantine, and inward-looking national policies and regulations in answer to a global phenomenon seem to stand in contrast to a global health perspective. Here, a higher education global health network can play a central role in reaching across borders to facilitate the exchange of experiences in times of shared hardship, fostering collaborations, strengthening partnerships, and to advocate for solidarity with those who are hit hardest, and to raise awareness of the importance of global health.

### Limitations

This study is not without limitations. While data are derived from a cross-sectional survey early on in the COVID-19 pandemic and our insights might not transfer to global health teaching in all contexts, this study synthesizes and compares insights from senior academic staff actively involved in global health teaching at 19 higher education institutes across the tropEd Network. It represents a unique snapshot and baseline of situations among higher education institutions in different settings and variations in severity of COVID-19 epidemics around the globe at the time. The potential for bias (e.g., social desirability bias, recall bias) is inherent in self-reported data, but was kept to a minimum through online data collection, and a common understanding of shared hardship. While we attempted to sample ‘in depth cases’ from diverse settings, the final sample size was ultimately also determined by constraints of time and resources. For example, the qualitative sub-sample of those who responded to the set of open-ended questions, did not include respondents from higher education institutions in Sub-Saharan Africa and Latin America. However, extensive discussions of the data and findings with tropEd members from various institutions suggested that the findings represented a wide range of perspectives from across the Network, and helped confirm the validity and reliability of the data. In addition, this study did not directly collect data on the students’ perspective, we are aware that this provider-centred approach may provide a skewed view on the impact of the pandemic.

## Conclusion

This international survey highlights that in order to reform and reconceptualise global health teaching and learning in times of COVID-19 and beyond, necessitates not only changes in didactic approaches, but also more fundamentally in terms of inclusivity, reciprocity, and solidarity across different settings worldwide.

## Supplementary Information

**Additional file 1.** Online Survey Questionnaire.

**Additional file 2.** Open Ended Questions.

## Data Availability

All data generated or analysed during this study are included in this published article.

## Abbreviations

GA: General Assembly
HEI: Higher Education Institution
tropEd: TropEd Network for Education in International Health
