# Supplementary material for: Adapting teaching and learning in times of COVID-19: a comparative assessment among higher education institutions in a global health network in 2020
Source: BMC Med Educ. 2022 Jun 28;22:507. doi: 10.1186/s12909-022-03568-4 (PMC9238047; doi:10.1186/s12909-022-03568-4)
Supplement: Supplementary file 1 — Additional file 1. Online Survey Questionnaire. [file 12909_2022_3568_MOESM1_ESM.pdf]

Changes and Challenges during Corona times: effects of the pandemic on International Health teaching among tropEd members

Form description

Email address *

Valid email address

This form is collecting email addresses. Change settings

Name of your institution *

Short answer text

1. How has your institution dealt with the Covid-19 outbreak at this moment? *

- [ ] Switched scheduled courses to online format
- [ ] Delayed the start dates of some courses
- [ ] Changed the application deadlines
- [ ] Changed the acceptance deadlines
- [ ] New offers
- [ ] Courses cancelled
- [ ] Modified recruiting process
- [ ] Adapted the selection process
- [ ] Students completed online course at tropEd partner institution
- [ ] Some practical courses move to next semester
- [ ] We want to still offers new courses this year
- [ ] more flexible on proof on english proficiency
- [ ] Other...

2. Has the spread of Covid-19 impacted your teaching and learning? *

Long answer text

How much does the following worry concern you? *

	very much	Much	Less	Not at all	I don't know
Sessions not h...	○	○	○	○	○
Staff mobility	○	○	○	○	○
Student mobility	○	○	○	○	○
Staff and stude...	○	○	○	○	○
Transformation...	○	○	○	○	○
Other (in this c...	○	○	○	○	○

Comment: *

Long answer text

4. The global health crisis will affect the plans of many students to study abroad. Could you tell us what you expect/believe regarding the Covid-19 pandemic in relation to the number of student applications you will receive? *

- ○ Increase the number of student applications
- ○ Decrease of the number of student applications
- ○ The number of student applications will remain the same

5. Which online communication strategies are you using currently to teach and to keep in contact with your students (e.g. Zoom, Skype, Microsoft Teams ....)? *

Long answer text

6. Which online communication strategies are you using currently to keep in contact with your international partners? *

Long answer text

7. What are the strategic keys responses your institution has taken to deal with the Covid-19 pandemic? *

Long answer text

8. How much has your institution adapted to distance learning in the Covid-19-pandemic?

- Very much
- Much
- Less
- Not at all
- I don't know

9. How well was your institution equipped in terms of digital infrastructure (online tools, capacitation of the teacher, etc.) to meet challenges in the Covid-19 pandemic?

- Very Much
- Much
- Less
- Not at all
- I don't know

10. Will your university implement social distancing while teaching in the Fall? *

- Yes
- No
- I don't know

10.1. If Yes, how?

Long answer text

11. How have you perceived the response of the tropEd secretariat with regard to the Covid-19 pandemic? Respond to the following statement: The response was timely and adequate *

- Strongly agree
- Agree
- Slightly agree
- Disagree
- I don’t know

12. How do you think the current situation will impact the network? *

- In a positive way
- In a negative way
- It will have no impact
- I don’t know

12.1. Why?

Long answer text

13. Which one do you think is the biggest challenge foreseen for the next academic year arising from the Covid-19 pandemic? *

Long answer text
