# Supplementary material for: Adapting teaching and learning in times of COVID-19: a comparative assessment among higher education institutions in a global health network in 2020
Source: BMC Med Educ. 2022 Jun 28;22:507. doi: 10.1186/s12909-022-03568-4 (PMC9238047; doi:10.1186/s12909-022-03568-4)
Supplement: Supplementary file 2 — Additional file 2. Open Ended Questions. [file 12909_2022_3568_MOESM2_ESM.pdf]

SARS-CoV-2 Survey tropEd

Please respond to the following 5 open-ended questions once per tropEd member institution

- Overview of COVID-19 impact on your institutions in general (patient care, expertise diverted to COVID-19, research activities, staff on sick-leave, quarantine, lockdown, etc.).

- Overview over your educational activities that would have been foreseen for the year 2020, and herein which ones are cancelled, which ones conducted as planned, which ones transferred into a new format (online, blended format, decreased SIT).

- Reaction on your COVID-19 responses by lecturers, applicants and participants.

- Outlook on 2021 and beyond, including building resilience for future comparable scenarios, restructuring of teaching activities.

- Suggestions for support in the post-COVID-19 (acute) phase by the tropEd network.

Thank You
